# Green Synthesis and Characterization of Gold Nanoparticles Using Lignin Nanoparticles

**DOI:** 10.3390/nano10091869

**Published:** 2020-09-18

**Authors:** Baobin Wang, Guihua Yang, Jiachuan Chen, Guigan Fang

**Affiliations:** 1 State Key Laboratory of Biobased Material and Green Papermaking, Qilu University of Technology (Shandong Academy of Sciences), Jinan 250353, China; wangbaobin0408@163.com (B.W.); chenjc@qlu.edu.cn (J.C.); 2 Institute of Chemical Industry of Forest Products, Chinese Academy of Forestry, Nanjing 210042, China

**Keywords:** lignin nanoparticles (LNPs), gold nanoparticles (Au NPs), green synthesis, nanotechnology, functional material

## Abstract

With the development of nanotechnology, gold nanoparticles (Au NPs) have attracted enormous attention due to their special properties. The green synthesis of Au NPs from lignin would inspire the utilization of lignin and its related functional materials. In this study, a rapid preparation process of Au NPs was investigated by utilizing lignin nanoparticles (LNPs) under room temperature without chemical addition. The LNPs acted as a reducing agent, stabilizing agent, and template for the preparation of LNPs@AuNPs. The obtained LNPs@AuNPs were characterized by UV-Vis spectrum, Transmission Electron Microscope (TEM), and X-ray photoelectron spectroscopy (XPS). The possible mechanism was illustrated by Fourier Transform Infrared Spectroscopy (FT-IR), ^31^P, XPS, and UV analyses. The abundant hydroxyl groups (24.96 mmol/g) favored the preparation of Au NPs. Au NPs diameters of 10–30 nm were well dispersed in the LNPs. The optimal reaction conditions were a ratio of 10 mg of LNPs to 0.05 mmol HAuCl_4_, room temperature, and a reaction time of 30 min. The LNPs@AuNPs exhibited excellent stability in the suspension for more than seven days. The reduction process could be related to the disruption of side chains of lignin, hydroxyl group oxidation, and hydroquinones and quinones from the comproportionation reaction. The LNPs@AuNPs would open a door for the design of Au NP/lignin-derived novel functional materials.

## 1. Introduction

With the development of nanotechnology and nanoscience, metal nanoparticles have attracted enormous attention because of their novel optical/electrical properties [1,2]. Metal nanoparticles, especially gold nanoparticles (Au NPs), are used as multifunctional gold nanoparticle-derived materials for various applications, including photothermal conversion [3,4], simultaneous detection and imaging [4,5], and targeted chemo-photothermal treatments [6], etc. Due to the versatility of the Au NPs, several strategies have been applied for the synthesis of Au NPs, such as irradiation reduction [7], thermal decomposition [8], and chemical reduction [9]. The most common method for the preparation of Au nanoparticles is chemical reduction. However, the chemical reduction process usually requires additional chemical addition (reducing agents, stabilizing agents, and surfactants) and harsh conditions which violate the basic principles of green chemistry [10]. Thus, a more environmentally friendly procedure is needed to prepare the Au NPs.

Lignin, the most abundant aromatic, is a biopolymer derived from trees and crops [11]. It is a three-dimensional amorphous polyphenol which consists of methoxylated phenylpropane substructures. Nowadays, millions of tons of industrial lignin are obtained from pulp mills; however, the lignin is normally used as fuel during the pulping process which is a rather inefficient way to utilize the lignin [11]. Extensive studies are devoted to exploit value-added applications, such as cosmetics, water treatment, adhesive, soil treatment, food, and medicine, etc. [12,13]. In the past decade, researchers found that the 3D structure and abundant surface groups (hydroxyl, carbonyl, and aldehyde groups) could act as reducing and stabilizing agents for the green synthesis of metal nanoparticles. Various approaches were applied in the green synthesis of metal nanoparticles including microwave irradiation, mechanical grinding method, and heat reduction. Han et al. reported Au NP-loaded liquid marble using lignin as a reducing agent under microwave radiation at 80 °C for 60 min [14]. The liquid marble exhibited excellent properties for the detection of Hg^2+^ and photothermal conversion. Lin et al. reported the green synthesis of Au NPs with the addition of hemicellulose/lignin at 100 °C [15]. With the development of material science, the regular structure of nanomaterials is essential to the design of functional materials for advanced applications. Moreover, the recent progress in the preparation of lignin nanoparticles (LNPs) inspired the design of lignin-based functional materials [16,17,18]. Thus, the green synthesis of well-dispersed Au NPs with LNPs is important for the preparation of composite lignin-derived materials.

Herein, LNPs were utilized as reducing and stabilizing agents for the green synthesis of well-dispersed LNPs@AuNPs under room temperature. The Au NPs were well dispersed in the LNPs due to the stabilizing effect of the lignin. Au NPs were confined in the LNP structure which would facilitate the design of the lignin-based materials. The total green synthesis of Au NPs was conducted under room temperature and without chemical addition which is consistent with the principles of green chemistry. Moreover, the reducing mechanism and reaction behavior were analyzed by ^31^P-NMR, FTIR, XPS, UV-Vis spectrum, and TEM. The possible mechanism was also proposed.

## 2. Materials and Methods

### 2.1. Materials

HAuCl_4_·3H_2_O was purchased from Sigma–Aldrich Co, Ltd. Ethanol (analytical grade) was purchased from Fisher, Co, Ltd. Lignin from prehydrolysis liquor was obtained through acid precipitation. The pH value of the prehydrolysis liquor was buffered to 2. Then, 10 g of prehydrolysis lignin (PL) was mixed with ethanol (300 mL) and stirred for 12 h. The solution was filtered, and the purified lignin was recovered by vacuum evaporation. All other reagents were used without further purification.

### 2.2. Preparation of LNPs

The LNPs were prepared following the anti-solvent method [19]. Specifically, 0.1 g lignin was added to 150 mL ethanol under magnetic stirring at 300 rpm for 2 h. Then, 70 mL deionized water was poured into the lignin/ethanol mixture, and the mixed solution was stirred for 4 h until the solution became a uniform solution. The solution was dialyzed in a dialysis bag (molecular weight: 12,000–14,000, Sigma–Aldrich Co, Ltd.) for seven days. The water was changed several times. Finally, the solution was freeze-dried for further characterization.

### 2.3. Preparation of Au NPs@ LNPs

LNPs (10 mg) were added to 10 mL of different concentrations of HAuCl_4_ (0.5 mmol/L, 2 mmol/L), and the mixture was sonicated in iced water for 10 min with magnetic stirring at 300 rpm. The obtained suspension was transferred to 20 mL glass vials under room temperature for 30 min. The samples were named LNPs@AuNPs 1 and LNPs@AuNPs 2, respectively. LNPs (10 mg) were added to 10 mL 0.5 mmol/L HAuCl_4_, and the mixture was sonicated in iced water for 10 min with magnetic stirring at 300 rpm. The obtained suspension was heated at 80 °C for 30 min in 20 mL glass vials. The sample was named LNPs@AuNPs 3. After the reaction completed, the suspension was centrifuged at 10,000 rpm for 10 min. The precipitation was washed with deionized water three times, and the obtained LNPs@AuNPs were used for further characterization. All the experiments were performed in duplicate.

### 2.4. Characterization

NMR analysis was processed with a Bruker AVIII 400 MHz spectrometer (Bruker, Karlsruhe, Germany) at room temperature. Phosphorous nuclear magnetic resonance (^31^P NMR) was used to quantitatively determine the functional groups of the lignin, and the detection of the subunits in lignin was performed through two-dimensional heteronuclear single quantum coherence (2D HSQC) NMR spectra. FTIR spectroscopy (NICOLET iS5, Thermo Fisher Scientific, Waltham, MA, USA) was used to characterize the chemical structure of lignin and the composites. The spectra were recorded in the range of 4000–500 cm^−1^ with a resolution of 4 cm^−1^ and 32 scans. The pH values were measured by a pH meter (ALTON pH510 Series). The morphology of LNPs and the composites was observed by a transmission electron microscope (TEM, JEOL 2011, Japan) with a 200 kV acceleration voltage. The samples were individually dispersed in water with sonication before being transferred to a carbon-coated copper grid and left to air-dry at room temperature overnight. Atomic force microscopy (AFM) was used to observe the morphology of LNPs. X-ray diffraction (XRD) patterns were recorded using a Bruker D8 Advance diffractometer with Cu Ka radiation (40 kV, 30 mA) at a scanning speed of 6°/min in the 2 θ range of 10–85°. The element composition of LNPs and the composites was performed using X-ray photoelectron spectroscopy (XPS) (ESCALa-b220i-XL Thermo Fisher Scientific K-Alpha, Thermo Fisher Scientific, Waltham, MA, USA). For the analysis of the XPS peaks, the C 1s and Au 4f position were set at 284.6 eV and 87.6 eV, respectively. Inductively coupled plasma atomic emission spectrometry (ICP-AES, Thermo Scientific ICAP 6500, Thermo Fisher Scientific, Waltham, MA, USA) was used to measure the gold content of composites after digestion with hydrochloric and nitric acid (3:1, *v/v*) [20]. The reduction process of Au NPs was recorded at room temperature using UV-Vis spectroscopy (Evolution 201, Thermo Fisher Scientific, Waltham, MA, USA). The characteristic peak of Au NPs is at 540 nm.

## 3. Results

### 3.1. Schematic of Preparing the LNPs@AuNPs

**Figure 1:**
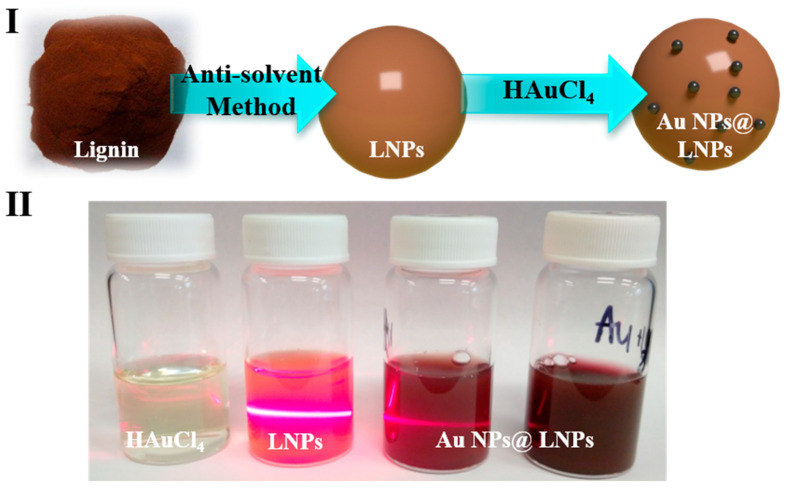
Schematics of the preparation of LNPs@AuNPs. (**I**). Preparation process of the LNPs@AuNPs, (**II**). Digital photo of the HAuCl_4_, LNPs, LNPs@AuNPs.

The LNPs@AuNPs were prepared through in situ reduction of gold nanoparticles onto LNPs under room temperature (Figure 1I). The PL-based LNPs were prepared through the anti-solvent method. The PL was dissolved in ethanol first, and then deionized water was added as an anti-solvent to obtain LNPs. The LNPs were formed due to intramolecular hydrogen bonding and π–π stacking of the lignin structure [21]. The green synthesis of Au NPs with LNPs was achieved by directly mixing HAuCl_4_ solution with LNPs. The Au NPs were reduced at room temperature with the reducing groups (hydroxyl and aldehyde groups) on the lignin structure (Figure 1II). With the increased concentration of HAuCl_4_ addition, the color of the LNPs@AuNP suspension turned red which is due to the SPR effect of the Au NPs. Moreover, LNPs acted as a stabilizer during the reduction process, and Au NPs were well dispersed in the LNPs. The Au NP suspension was stable even after seven days, which is due to the electrostatic repulsion of the negatively charged surface of the LNPs.

### 3.2. Characterization of Lignin

**Figure 2:**
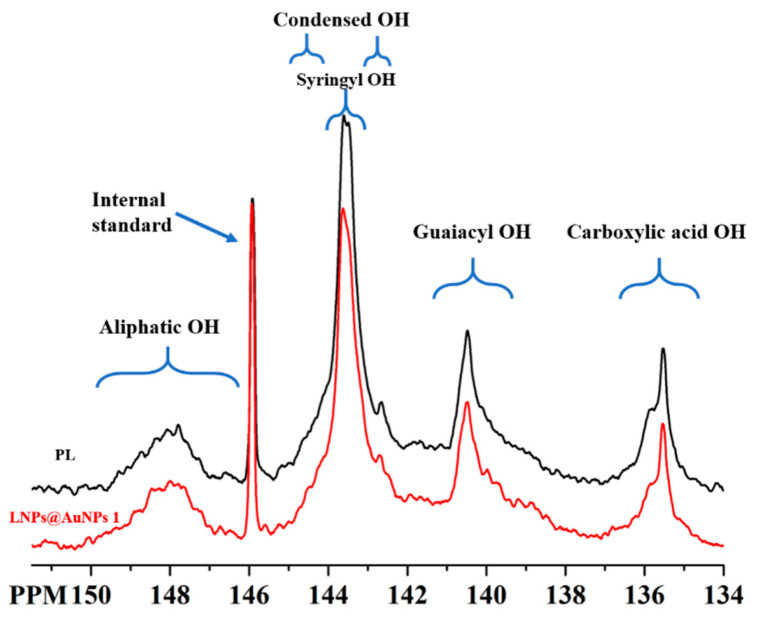
^31^P of the PL and LNPs@AuNPs 1.

The green synthesis of Au NPs was related to the surface functional groups of the lignin; thus, it is essential to analyze the chemical structure of the lignin. The chemical structure of the lignin was characterized with FTIR and ^31^P spectra. Figure 2 shows the ^31^P spectrum of the PL. The main OH groups (syringyl and guaiacyl –OH) were observed in the PL which is the typical structure of lignin. The total –OH, noncondensed phenolic –OH and carboxylic acid contained 24.96, 14.47 and 2.34 mmol g^−1^ lignin, respectively (Table 1). After the reduction process, the content of the aliphatic and phenolic hydroxyl groups decreased, and the content of the carboxylic groups increased. The results indicated the reduction process may be related to the oxidation of hydroxyl groups. The abundant noncondensed phenolic hydroxyl groups favored the preparation of Au NPs.

**Figure 3:**
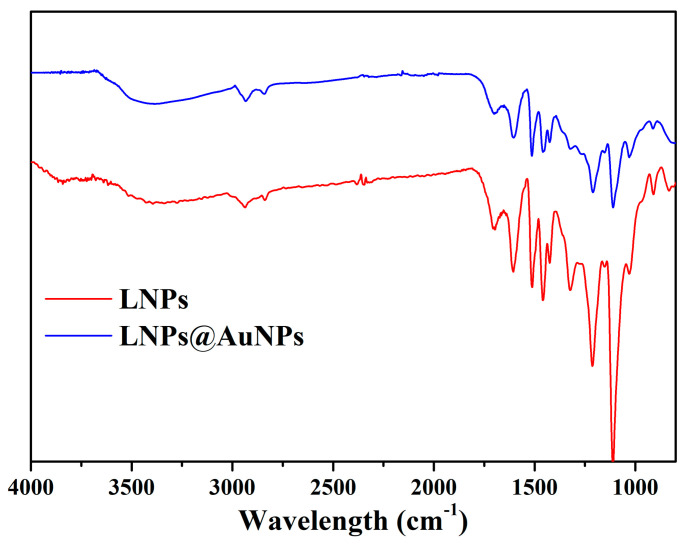
FTIR spectra of the LNPs and LNPs@AuNPs.

FTIR was used to further characterize the chemical structure of the LNPs and LNPs@AuNPs (Figure 3). The characteristic peak at 3300 cm^−1^ can be assigned to the phenolic hydroxyl and aliphatic hydroxyl groups of the lignin structure. The peak at 3000–2800 cm^−1^ is related to the methyl and methylene groups [14]. The peak around 1600–1500 cm^−1^ can be assigned to the vibration of benzene rings. The peak in the range of 1300–1200 cm^−1^ is related to C–O/C=O groups. Also, the peak at 1660 cm^−1^ is related to the conjugated carbonyl groups. The peak intensity of hydroxyl groups (3300 cm^−1^, 1212 cm^−1^) decreased after the reduction process which indicated the important role of hydroxyl groups in the preparation of Au NPs. These results demonstrated that LNPs possessed abundant hydroxyl and aldehyde groups which guaranteed the successful preparation of Au NPs [22]. The minor shift of hydroxyl groups that occurred with the LNPs@AuNPs showed the interactions between LNPs and Au NPs.

**Figure 4:**
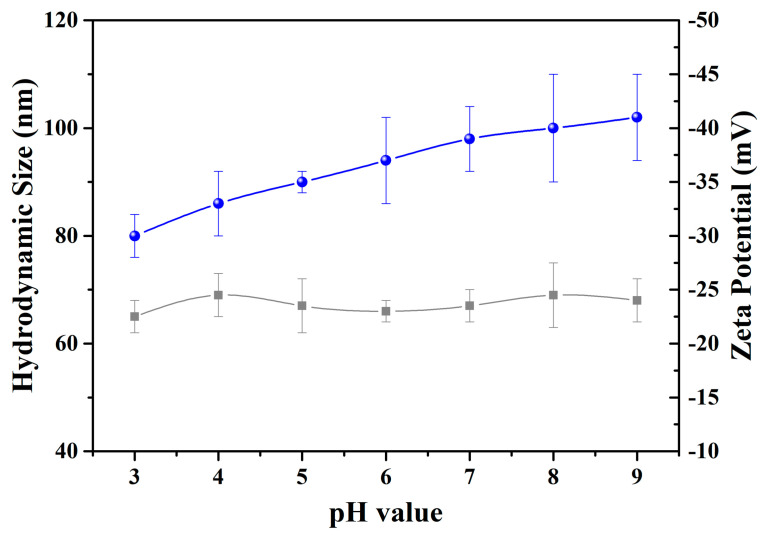
Variation of hydrodynamic size and zeta potential of LNPs with different pH values.

**Figure 5:**
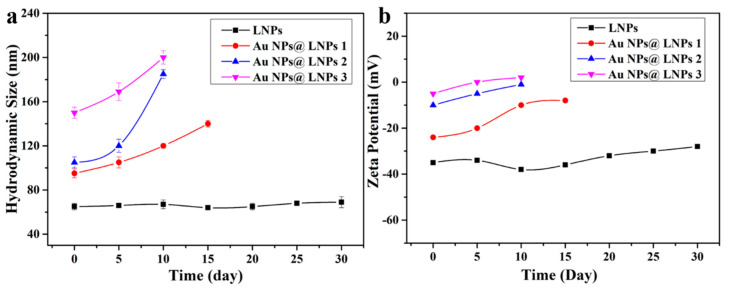
Variation of hydrodynamic size (**a**) and zeta potential (**b**) of LNPs and the composites during long-term storage.

Figure 4 shows the hydrodynamic size and zeta potential of the LNPs with different pH values. DLS results demonstrated that LNPs were stable with a large pH range, and the hydrodynamic size of the LNPs was around 70 nm. The zeta potential of the negatively charged LNPs increased with the increase in pH which is due to the deprotonation process. Figure 5 shows the stability of the LNPs during long-term storage. The LNPs were stable over 30 days. The composites aggregated gradually after 10 days due to the decreased zeta potential which might be related to the association between the negatively charged LNPs and ionic species in the aqueous media.

### 3.3. UV-Vis Spectrum of the Preparation of LNPs@AuNPs

**Figure 6:**
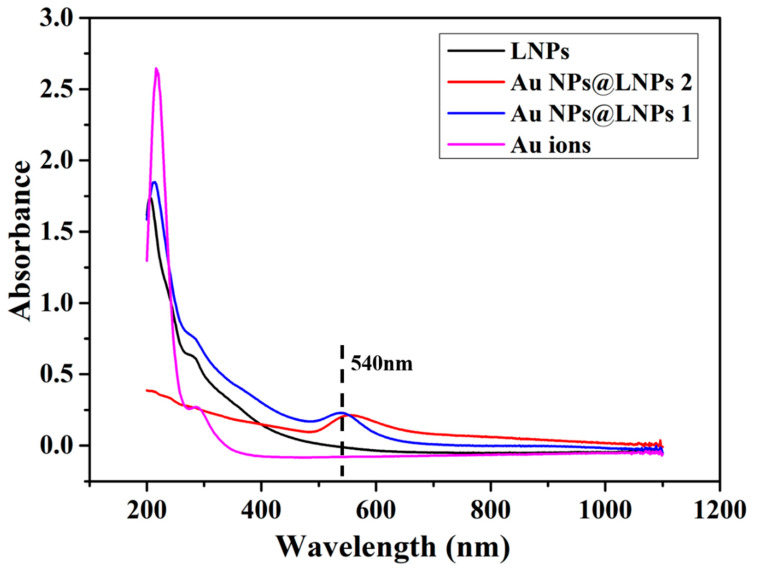
UV-Vis spectra of the LNPs, Au ions and LNPs@AuNPs.

Lignin was utilized as a reducing agent and stabilizing agent for the preparation of Au NPs. LNPs enabled the well-ordered structure of the nanocomposites. UV-Vis spectra were used to monitor the preparation of Au NPs (Figure 6). The LNPs were formed due to hydrogen bonding, van der Waal’s forces, electrostatic interaction, and π–π interactions. The adsorption band at 280 nm is the characteristic band of lignin [23]. The adsorption of nanocomposites demonstrated a red shift indicating the interaction between metal ions and negatively charged LNPs. The LNPs@AuNPs showed the strong characteristic of the SPR adsorption band of Au NPs at 540 nm which confirmed the successful preparation of Au NPs (LNPs@AuNPs 1). With the increase in the concentration of HAuCl_4_, the characteristic adsorption band showed a blue shift, indicating that the size of the nanoparticles decreased (LNPs@AuNPs 2) [24].

## 4. Discussion

### 4.1. TEM of LNPs and Au NPs@ LNPs

**Figure 7:**
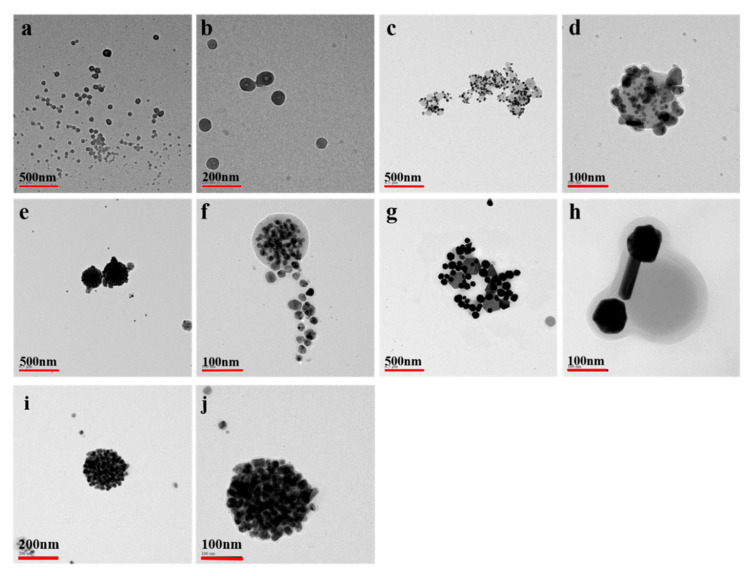
TEM of the LNPs (**a**,**b**), LNPs@AuNPs 1 (**c**,**d**), LNPs@AuNPs 2 (**e**,**f**), LNPs@AuNPs 3 (**g**,**h**), Au NPs@ LNPs 2 storage for seven days (**i**,**j**).

TEM was used to further verify the morphology of the LNPs and LNPs@AuNPs. The reduction process and morphology variation of the LNPs were monitored by TEM. Figure 7a,b shows the morphology of the LNPs. The LNPs demonstrated two main structures: yolk shell structure and hollow structure. According to statistics, the size of the yolk shell LNPs occupied 77% of the total LNPs with a size below 70 nm, while the hollow LNPs occupied 23% of the total LNPs with a size between 80 and 100 nm.

The Au NPs were synthesized in situ by using the hydroxyl and aldehyde groups of the LNPs. Figure 7c,d shows the morphology of the LNPs@AuNPs. The diameter of the gold nanoparticles was in the range of 5–30 nm. The well-defined structure of the LNPs and well-dispersed gold nanoparticles verified that LNPs could act as excellent reducing agents and capping agents for the preparation of gold nanoparticles. Figure 7e,f demonstrates that the increased HAuCl_4_ concentration led to decreased average gold nanoparticles. However, the increased HAuCl_4_ concentration resulted in the collapse of the hollow structure of LNPs. Part of gold nanoparticles are from the LNPs. Thus, a moderate HAuCl_4_ concentration should be controlled. Figure 7g,h indicates the Au NPs prepared at 80 °C for 30 min. The increased temperature led to the inferior control of the growth of gold nanoparticles. Furthermore, the Au NPs@ LNP composites exhibited excellent dispersal ability after storage for seven days (Figure 7i,j). These results verified that well-dispersed gold nanoparticles with well-defined lignin nanoparticles could be prepared at room temperature with the concentration of HAuCl_4_ at 0.5 mmol/L.

ICP-AES was used to quantify the loading of Au NPs onto the LNPs. The Au NP loading for LNPs@AuNPs 1, LNPs@AuNPs 2, and LNPs@AuNPs 3 was 4.59%, 12.54%, and 8.59%, respectively. The loading of Au NPs increased with the increase in precursor concentration and temperature.

### 4.2. XPS of LNPs and Au NPs@ LNPs

**Figure 8:**
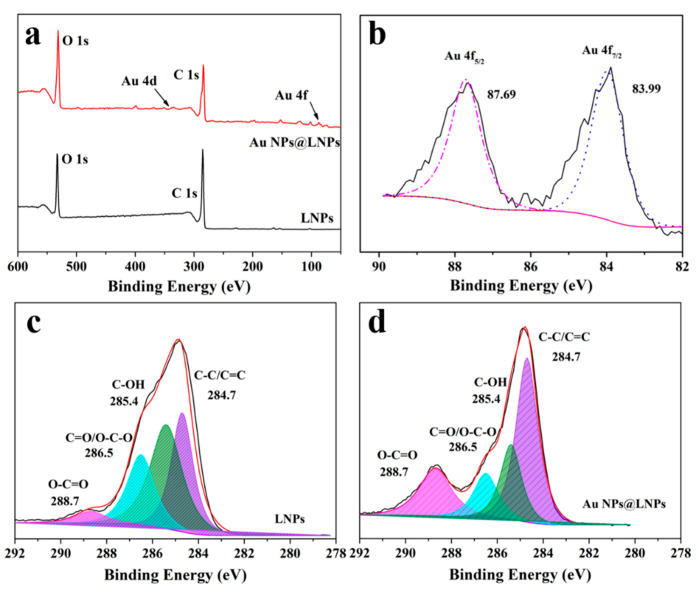
(**a**) XPS of the wide scan of LNPs and Au NPs@ LNPs. (**b**) Au 4f of the Au NPs@ LNPs (**c**) C 1s of the LNPs, (**d**) C 1s of the Au NPs@ LNPs.

XPS wide scans showed the signals of carbon and oxygen (Figure 8a). The LNPs@AuNPs exhibited the signals of Au which further confirmed the successful preparation of gold nanoparticles (Figure 8b). The C 1s spectra of LNPs and LNPs@AuNPs with their deconvolutions are shown in Figure 8c,d, respectively. The deconvolution of LNPs and LNPs@AuNPs on C 1s shows four similar peaks: peak 1 with binding energy 284.7 eV is related to a carbon bound to carbon atoms (C–C) or carbon–carbon double bond (C=C), peak 2 at 286.5 eV corresponds to a carbon atom bound to a non-carbonyl oxygen atom (C–OH), peak 3 at 285.4 eV represents a carbon atom bound to one carbonyl oxygen atom (C=O) or two non-carbonyl oxygen atoms (O–C–O), and peak 4 at 288.6 eV corresponds to peaks for O–C=O. Compared with the LNPs, the bands signal of C=O and O-C=O are stronger, while the signal of C–O decreased obviously. Thus, the side chain of lignin was disrupted during the reduction process, and the quinone structure or carboxylic groups were formed [25].

### 4.3. Proposed Mechanism for the Preparation of LNPs@AuNPs

The green synthesis of gold nanoparticles is related to electrostatic interaction and the reduction effect due to hydroxyl and aldehyde groups in the lignin structure. Adsorption and reduction were completed first on the surface of the LNPs, and large metal nanoparticles formed. As the reaction proceeded, the Au^3+^ was adsorbed inside the LNPs, the gold nanoparticles formed slowly inside the LNPs, and the gold nanoparticles were small and well dispersed due to the stabilizing/capping effect.

**Figure 9:**
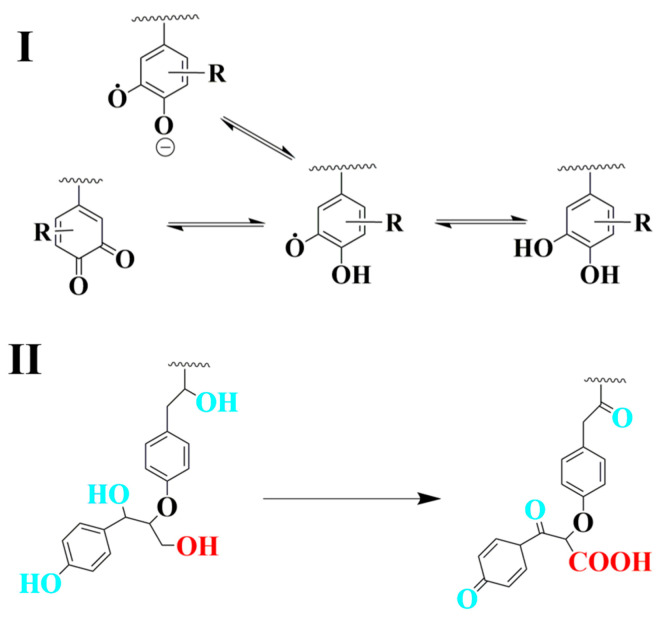
Proposed mechanism for the preparation of gold nanoparticles: (**I**) quinone related reduction mechanism, (**II**) hydroxyl groups related reduction mechanism.

Lignin contains stable radicals at room temperature. The main radicals have a semi-quinone structure [25]. During the reduction process, the hydroquinones and quinone structure of lignin formed due to the comproportionation reaction of semiquinone radicals, and semiquinone radical anions might be related to the formation of gold nanoparticles (Figure 9I). Also, the abundant phenolic groups favor the reduction of Au NPs. The surface hydroxyl groups would be transferred to carbonyl/carboxylic groups which contribute to the preparation of Au NPs (Figure 9II).

## 5. Conclusions

To achieve total green synthesis of Au NPs with lignin, we reported the preparation of LNPs@AuNPs by using LNPs without chemical addition at room temperature. The LNPs acted as a reducing agent, stabilizing agent, and template for the preparation of LNPs@AuNP nanocomposites. The abundant noncondensed hydroxyl groups (11.49 mmol/g) favored the preparation of Au NPs. The Au NP diameters of 1–30 nm were well dispersed in the LNPs. The optimal reaction conditions were a ratio of 10 mg of LNPs to 0.05 mmol HAuCl_4_, room temperature, and a reaction time of 30 min. The Au NPs@ LNPs exhibited excellent stability in the suspension for more than seven days. The reduction process could be related to (i) disruption of side chains of lignin, hydroquinones, and quinones from the comproportionation reaction, (ii) the surface hydroxyl groups would be transferred to carbonyl/carboxylic groups, and (iii) abundant noncondensed phenolic groups. The LNPs@AuNPs would inspire the design of LNPs@AuNPs derived from functional materials for application as sensors, responsive material, and actuators.

## Figures and Tables

**Table 1:** Quantitative analysis of ^31^P of PL.

Samples	Aliphatic OH	Condensed OH	Syringyl OH	Guaiacyl OH	Carboxylic OH
PL	2.54	5.78	8.66	5.81	2.17
LNPs@AuNPs	2.36	4.97	6.37	4.10	2.34
